# Mercapto-Functionalized Porous Organosilica Monoliths Loaded with Gold Nanoparticles for Catalytic Application

**DOI:** 10.3390/molecules24234366

**Published:** 2019-11-29

**Authors:** Hongwei Li, Junhui Pan, Chengtao Gao, Mengyu Ma, Liangyu Lu, Yuzhu Xiong, Fuping Dong

**Affiliations:** 1 Department of Polymer Materials and Engineering, Guizhou University, Guiyang 550025, China; lhwnoonoo123@163.com (H.L.); 18275569503@163.com (J.P.); chengtaogao@163.com (C.G.); mmymamengyu@163.com (M.M.); gs.lylu17@gzu.edu.cn (L.L.); yzxiong@gzu.edu.cn (Y.X.); 2 National Engineering Research Center for Compounding and Modification of Polymer Materials, Guiyang 550025, China

**Keywords:** porous organosilica monolith, silicone-based materials, organic–inorganic hybrid materials, gold nanoparticles, sol-gel, catalyst

## Abstract

Porous organosilica monoliths have attracted much attention from both the academic and industrial fields due to their porous structure; excellent mechanical property and easily functionalized surface. A new mercapto-functionalized silicone monolith from a precursor mixture containing methyltrimethoxysilane; 3-mercaptopropyltrimethoxysilane; and 3-mercaptopropyl(dimethoxy)methylsilane prepared via a two-step acid/base hydrolysis–polycondensation process was reported. Silane precursor ratios and surfactant type were varied to control the networks of porous monolithic gels. Gold nanoparticles were loaded onto the surface of the porous organosilica monolith (POM). Versatile characterization techniques were utilized to investigate the properties of the synthesized materials with and without gold nanoparticles. Scanning electron microscopy was used to investigate the morphology of the as-synthesized porous monolith materials. Fourier transform infrared spectroscopy was applied to confirm the surface chemistry. ^29^Si nuclear magnetic resonance was used to investigate the hydrolysis and polycondensation of organosilane precursors. Transmission electron microscopy was carried out to prove the existence of well-dispersed gold nanoparticles on the porous materials. Ultraviolet–visible spectroscopy was utilized to evaluate the high catalytic performance of the as-synthesized Au/POM particles

## 1. Introduction

Porous monoliths materials, including silica [1], grapheme [2], carbon nanotubes [3], and cellulose [4,5,6], have attracted much attention for versatile application due to their unique properties of low density, thermal conductivity and a high adsorption capacity [7,8,9,10]. Porous organosilica monoliths (POM), as one kind of molecular level organic/inorganic hybrid materials, are a class of silica-based materials containing organic groups as integral parts of their structures [11]. These hybrid materials could offer a reactive and easily-modified surface, large pore size and higher mechanical strength than conventional silica. They have had attractive applications in the field of catalysts [12,13,14,15], drug carriers [16], oil/water separation [17], heavy metal removal [18,19], chromatographic separations [20], and reverse osmosis desalination etc. [21]. The porous structure could be obtained from the hydrolysis and condensation of difunctional or trifunctional alkoxysilanes.

In recent years, the functionalized organosilica materials with different organic groups (methyl, epoxide, amine and mercaptopropyl) on the surface have been studied [22,23,24,25,26,27]. Zhang et al. fabricated the amine-functionalized porous organosilica monoliths with rich and adjustable pore structure via a one-step emulsion-templated process [18]. This amino-POMs demonstrated excellent Cr(VI)-removal capacity with the adsorption efficiency to be 92.8% at room temperature, and good durability with the efficiency still remaining as high as 85.1% after five recycles. Kanamori et al. fabricated methyl-functionalized marshmallow-like gels derived from tri- and difunctional alkoxysilanes as co-precursors through a facile one-pot reaction [28]. The obtained methyl-functionalized gels display superhydrophobicity and could work as a sponge to remove organic compounds from water by absorbing them and releasing them upon being squeezed out. In our previous reports, the hydrolysis–condensation of methytrimethoxysilane and mercaptopropyltrimethoxysilane (SHTMS) silane co-precursor could form porous core–shell structures by using polystyrene as a structure-directing agent [29]. After the loading of gold nanoparticles, the porous nanocomposite materials showed excellent catalytic performance.

In this work, a new mercaptopropyl-functionalized monolith was fabricated via a facile process by using an aqueous solution containing alkoxysilane co-precursors, acetic acid (HAc), urea, and surfactant. The final monolith materials were obtained after heating at 95 °C for 12 h, washing with water, and drying under an ambient condition. The POMs were controlled by altering the starting silane precursor composition and surfactant type. The morphology, microstructure, chemical composition, and thermal stability of the resultant monolith materials were investigated by the techniques of scanning electron microscopy (SEM), Fourier transform infrared (FTIR) spectroscopy, ^29^Si nuclear magnetic resonance (NMR), thermal gravimetric analysis (TGA), and elemental analysis (EA). Gold nanoparticles were immobilized into the pores of the porous monolith materials due to the high affinity between the -SH groups and Au ions. Au/POMs nanocomposite particles showed high catalytic performance with the reduction of 4-nitrophenol (4-NP) as a model reaction.

## 2. Results

**Figure 1:**
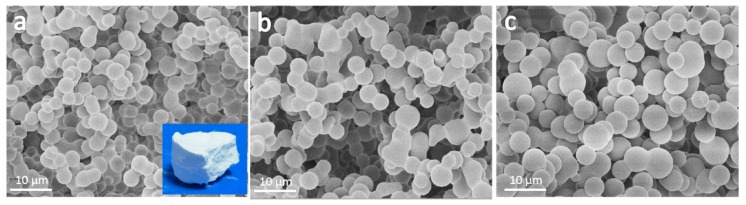
SEM images of the porous organosilica monoliths fabricated from silane co-precursor with different volume ratios of MTMS: SHTMS: SHDMS, which were as follows: (**a**) 3:1:2, (**b**) 2:1:2 and (**c**) 1:2:2.

The POMs were fabricated via a sol–gel process with methyltrimethoxysilane (MTMS), mercaptopropyltrimethoxysilane (SHTMS) and 3-mercaptopropyl(dimethoxy)methylsilane (SHDMS) as co-precursor, acetic acid and urea as catalysts, water as a solvent, hexadecyltrimethylammonium bromide (CTAB) as a surfactant. The hydrolysis-polycondensation reaction of silane co-precursors were catalyzed by the dilute acetic acid and at the same time the urea was hydrolyzed into ammonia and CO_2_ above 60 degrees, which raised the pH value and accelerated the condensation reaction. The final sponge-like samples were obtained as shown in the inset images in Figure 1a. The connected microspheres were observed at the cross-section surface and the porous structures were observed clearly for all samples, as the SEM images illustrated in Figure 1. The pores of the sample were formed by the accumulation of closely connected microspheres, which had high formability, a smooth surface, and uniform size.

The composition of the silane precursor played an important role in controlling the morphology of the samples. The tightly connected microspheres were observed in all samples. When the volume ratio of MTMS: SHTMS: SHDMS changed from 3:1:2 to 1:2:2, the average particle size of microspheres increased from about 3.1 to 4.4 μm. This phenomenon was due to the high hydrolysis–condensation speed of methyltrymethoxysilane and the large steric hindrance of the mercapto group. EA was performed to estimate the mercaptopropyl group content on the sample, and results showed that the S content in the sample increased from 16.3% to 21.5%.

**Figure 2:**
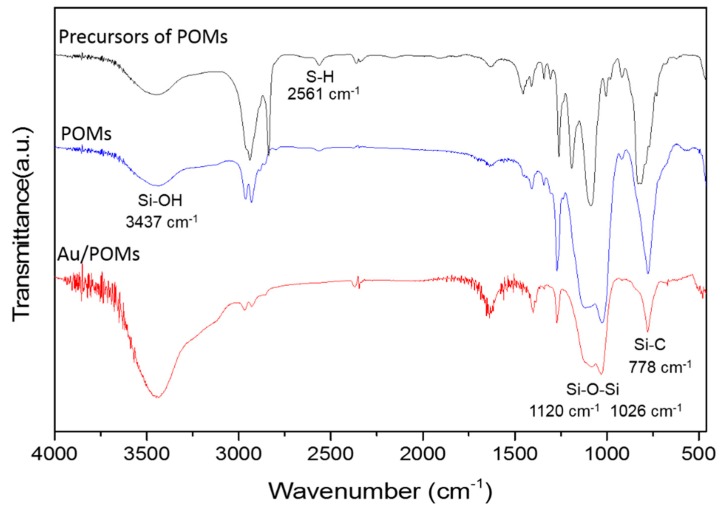
Fourier transform infrared (FTIR) spectra of the porous organosilica monoliths with and without Au as well as the spectrum of the precursor of POMs-1 (MTMS/SHTMS/SHDMS = 3:1:2).

Figure 2 shows the Fourier transform infrared (FTIR) spectra of the POMs with and without Au, as well as of the precursors of POMs (MTMS/SHTMS/SHDMS = 3:1:2). In the spectra of the POMs with and without Au, two strong absorption peaks at 1120 and 1026 cm^−1^ represented the Si–O–Si structure of POMs (Figure 2) [30]. These peaks did not appear in the spectrum for the precursors of POMs. In all spectra, the peaks at 1271 cm^−1^ for the C–H group in the SiCH_3_ group and 778 cm^−1^ for the Si–C group demonstrated that the methyl group was connected to the Si atom directly [31]. The peak at 2561 cm^−1^ for the SH group were observed in POMs, which proved the successful formation of mercaptopropyl-functionalized POMs [32]. The same peak at 2561 cm^−1^ for the SH group became weaker after loading Au nanoparticles due to the interaction between thiols and gold surfaces.

**Figure 3:**
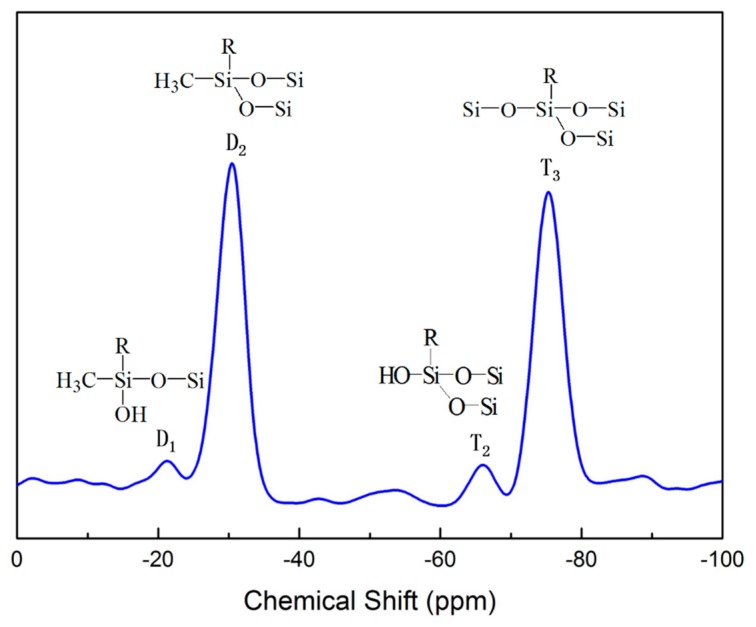
Solid ^29^Si nuclear magnetic resonance (^29^Si NMR) spectrum of porous organosilica monoliths with a MTMS/SHTMS/SHDMS = 3:1:2 composition.

Figure 3 shows the ^29^Si CP-MAS NMR spectrum of the porous organosilica monoliths with a MTMS/SHTMS/SHDMS composition ratio of 3:1:2. In the spectrum both the monosubstituted silica (T) and disubstituted silica (D) species were clearly visible. Because the SH-DMS had a RSi(CH_3_)(OCH_3_)_2_ structure, the resultant Si-O linkage formed by hydrolysis afforded the Dn units. On the other hand, the RSi((OCH_3_)_3_) from MTMS and SHTMS formed the Tn units. The strong peaks at −30 ppm and −75 ppm represented the D_2_ (RSi(OSi) OSi) and T_3_ (SiOSi(OSi)OSi) units respectively, which demonstrated the complete condensation of silane precursors [33,34]. The relatively weak signals at −21 ppm and −66 ppm were assigned to the D_1_ and T_2_ units due to the incomplete condensed silanes, which demonstrated that almost all silane precursors had been fully condensed [35]. The cross-linked Si-O-Si framework could be confirmed in the porous organosilica monoliths due to the sol-gel hydrolysis and condensation of Si-OCH_3_.

**Figure 4:**
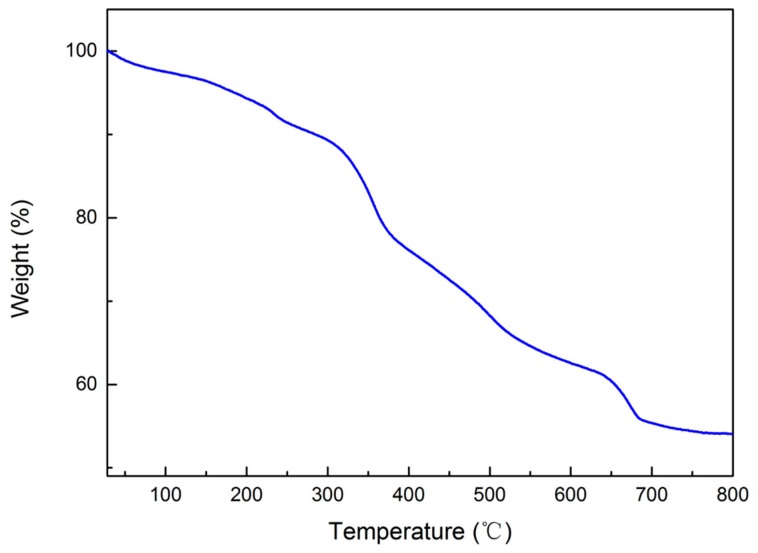
Thermal gravimetric analysis (TGA) data of the porous organosilica monoliths with a MTMS/SHTMS/SHDMS = 3:1:2 composition.

As shown by the TGA data in Figure 4, the weight loss curve presented a slow decrease at <250 °C due to water adsorption [36]; showed a rapid decrease between 250 °C and 700 °C due to the decomposition of the mercaptopropyl and methyl groups on the microsphere surface, and illustrated a steady level, with the remaining weight of 56.78% at > 700 °C. These results indicated the stability of the materials due to the existence of the inorganic Si-O-Si framework [37].

**Figure 5:**
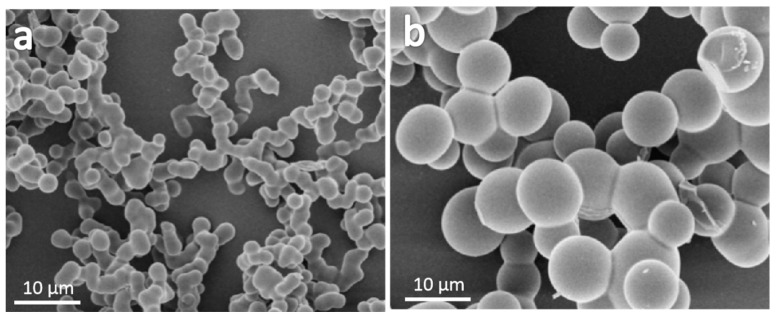
SEM images of POMs with different surfactants (**a**) hexadecyltrimethylammonium chloride (CTAC) and (**b**) sodium lauryl diphenyl ether disulfonate (SLD).

The surfactant type also plays an important role in the formation of POMs and can control the morphology of the final samples. POMs with a size of 2.6 µm can be obtained with connecting microspheres when utilizing hexadecyltrimethylammonium chloride (CTAC) as the surfactant (Figure 5a), whereas POMs with a size of 8.0 µm can be obtained when utilizing sodium lauryl diphenyl ether disulfonate (SLD) as surfactant (Figure 5b).

**Figure 6:**
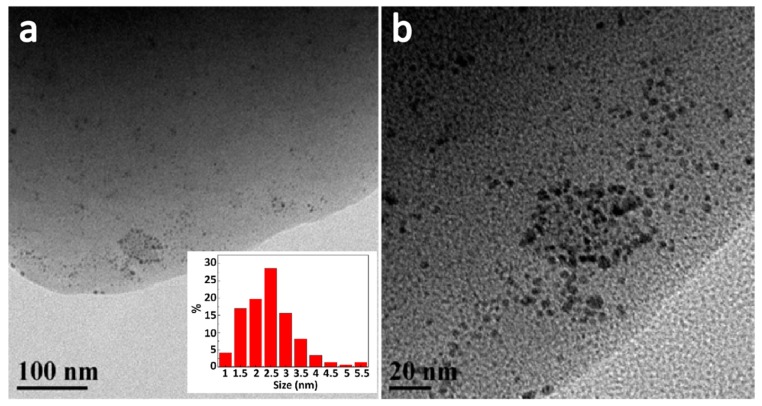
TEM images of the organosilica monolith particles with a MTMS/SHTMS/SHDMS = 3:1:2 composition loaded with gold nanoparticles at (**a**) low magnification (inset is the corresponding size distribution of gold nanoparticles) and (**b**) high magnification.

The porous structure and the mercaptopropyl-functionalized surface make the materials suitable for metal ion adsorption. In this work, gold nanoparticles were loaded onto the surface of the grounded POM powders with average particle size of 3.1 μm by using HAuCl_4_ as the gold source and NaBH_4_ as the reducing agent. As shown in the HRTEM image (Figure 6), dark globular spots can be observed, which confirmed that gold nanoparticles were successfully loaded on POM particles. The estimated average diameter of the gold nanoparticles was 2.7 nm, and no distinct aggregation was observed. Inductively coupled plasma (ICP) analysis revealed that the gold content was 0.68 wt.%, which also proved that gold nanoparticles were successfully loaded onto the sample.

**Figure 7:**
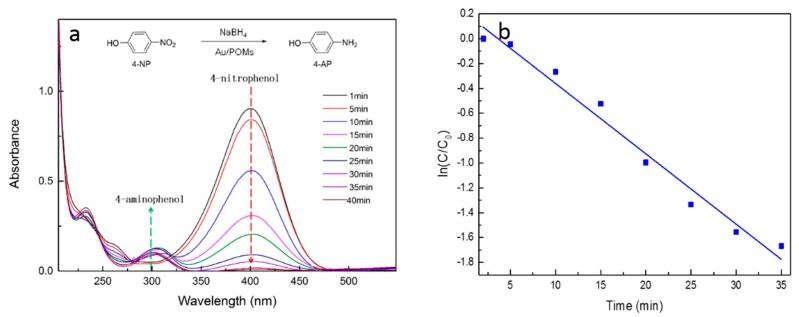
(**a**) Ultra Violet-Visible spectra of 4-nitrophenol reduction with Au/PSMs-1 as a catalyst and (**b**) plot of ln(C/C_0_) versus time using Au/POMs-1 as the catalyst.

The catalytic performance of Au/POM nanocomposite particles were determined with the reduction of 4-nitrophenol (4-NP) to 4-aminophenol (4-AP) in the presence of NaBH_4_ as the model reaction. Without utilizing Au/POM nanocomposite particles as the catalyst, no significant color change, which indicated that the reduction of 4-NP would occur. When Au/POMs were used as catalysts, the characteristic absorption peak of *p*-nitrophenol at 400 nm gradually weakened with reaction time (Figure 7a). At the same time, a new peak appeared at approximately 300 nm, which was the amino peak of 4-AP formed by nitrophenol reduction [38]. After a 40 min reaction, the suspension became completely colorless, which indicated the complete reduction of 4-NP. Figure 7b showed the ln(C/Co) versus reaction time for the reduction of 4-NP over Au/POMs nanocomposite. The kinetic constant k_app_ was 0.0566 min^−1^, which was similar to other substrate-supported Au nanocatalysts [39,40]. The AuNPs were well dispersed in the pores of POMs, allowing effective contact with 4-NP and demonstrated a good catalytic efficiency. The cyclability of Au/POM particles was also investigated using the same catalytic reaction. A 4-NP conversion rate of > 96.3% was obtained after five runs, proving that the existence of POM particle support was enough to stabilize the catalytic nanoparticles by blocking their aggregation.

## 3. Materials and Methods

### 3.1. Chemicals

Methyltrimethoxysilane (MTMS, 98%), mercaptopropyltrimethoxysilane SHTMS (95%), 3-mercaptopropyl(dimethoxy)methylsilane (SHDMS, 95%), urea (99.5%), hexadecyl trimethyl ammonium bromide (CTAB, 99%), hexadecyltrimethylammonium chloride (CTAC, 97%), HAc (99.5%), ethanol (99.7%), HAuCl_4_ (≥99.9%), and 4-NP (99%) were purchased from Aladdin (Shanghai, China). NaBH4 (98%) was supplied by the Tianjin Kermel Chemical Reagent Co., Ltd. (Tianjin, China). Sodium lauryl diphenyl ether disulfonate (SLD) was obtained from Shanghai Aichun Biological Technology Co., Ltd. The water used throughout the experiment was distilled water (~17 MΩ) produced by the Milli-Q water system.

### 3.2. Synthesis of Porous Organosilica Monoliths (POMs)

Typically, CTAB (1 g) and urea (5 g) were added to 15 mL diluted HAc solution and stirred for 30 min. Then, the organosilane co-precursor, which contained different amounts of MTMS, SHTMS, and SHDMS (Table 1), was added into the system and stirred at room temperature for 1 h. Afterward, the vial was placed in an oven at 95 °C for 12 h. The obtained gel was cooled naturally and washed thrice with water and ethanol. To investigate the effect of surfactant on the morphology of the monoliths, CTAC and SLD were utilized to replace CTAB with the same conditions as the sample POMs-1.

### 3.3. Porous Organosilica Monoliths Loaded with Gold Nanoparticles

Au/POM composites were fabricated through an impregnation process with grounded POM particles as the carrier and HAuCl_4_ as the gold source. HAuCl4·3H_2_O solution (10 mL, 10 mM) was added to 10 mL POM suspension (containing 0.025 g POM solid, 5 mL water and 5 mL ethanol) and stirred at room temperature for 12 h. Then, for the system, a considerable amount of newly prepared NaBH_4_ solution was added and stirred for 3 h. Samples were collected by filtration and dried in a vacuum oven at 60 °C for 3 h for further use.

### 3.4. Catalytic Reduction of 4-Nitrophenol with Au/POMs as the Catalyst

1 mL of Au/POMs ethanol suspension (1 mg/mL) was mixed with 1.5 mL newly prepared aqueous NaBH4 solution (7 mM), and the mixture was added with 1.5 mL 4-NP (0.14 mM) and stirred until the bright yellow-colored mixture gradually became colorless. The reaction progress was monitored by obtaining the ultraviolet–visible (UV–Vis) absorption spectrum of the system. The particles were filtered after each reaction to study catalyst recycling. The particles were filtered, collected carefully, cleaned thoroughly with water, and dried in an oven for 6 h at 60 °C. Then, under the same reaction conditions, the catalyst was reused for subsequent reaction.

### 3.5. Characterizations

The morphology of the POMs was obtained by scanning electron microscopy using the FEI-SEM system (FEI Helios Nanolab 600i, FEI, Hillsboro, OR, USA) at 5 kV. The surface chemistry was determined by FTIR spectroscopy using the Perkin–Elmer Spectrum GX-spectrophotometer (Thermo, Waltham, MA, USA) with a spectral resolution of 1 cm^−1^ and scan number of 32. The molecular structure was confirmed by the ^29^Si NMR technique using the Varian Inc. 400 MHz UNITY INOVA spectrometer (Varian Medical Systems, Palo Alto, CA, USA) at room temperature and magic-angle spinning, resonance frequencies, and 90° pulse length of 5 kHz, 79.5 MHz, and 6.5 µs, respectively. The morphology of the gold nanoparticles on POMs were observed using the FEI Tecnai G2F30 electron microscope (FEI) operating at 200 kV. For the nanoparticle size gauge values, the average of ≥100 particles on the TEM images were taken. The UV–Vis spectra were obtained using the Evolution 201 (Thermo) UV–vis spectrophotometer with 1 cm quartz cuvettes. TGA was conducted using the Mettler TGA/DSC 3+ (Mettler-Toledo, Zurich, Switzerland) from natural temperature to 800 °C at a heating rate of 5 °C min^−1^ under N_2_ atmosphere. EA was performed using the Vario EL Cube (Elementar, Frankfurt, Germany) EA instrument. Inductively coupled plasma (ICP) analysis was performed using the Perkin-Elmer Nexion 300 (Perkin-Elmer, Waltham, MA, USA).

## 4. Conclusions

In summary, mercapto-functionalized POMs without post-synthesis functionalization were prepared via a sol–gel process. The SEM images showed that the POMs had rich highly adjustable porous structures that were connected to the microsphere structure via a simple change in the formulation of the silane precursor. The successful graft of the mercapto groups on the surface of the organosilica monolith was confirmed by FTIR spectroscopy and EA. The POM particles prepared in this work showed excellent catalytic performance after loading with gold nanoparticles. The mercapto-functionalized POMs can be produced in a large scale and has potential as a catalyst and in the adsorption field.

## Figures and Tables

**Table 1:** Synthesis of porous organosilica monoliths with different parameters.

Code	CTAB (g)	Urea (g)	HAc (mL)	MTMS (mL)	SHTMS (mL)	SHDMS (mL)
POMs-1	1	5	15	3	1	2
POMs-2	1	5	15	2	1	2
POMs-3	1	5	15	1	2	2

## References

[1] Maleki H., Whitmore L., Husing N. (2018). Novel multifunctional polymethylsilsesquioxane-silk fibroin aerogel hybrids for environmental and thermal insulation applications. J. Mater. Chem. A.

[2] Xiao J.L., Tan Y.Q., Song Y.H., Zheng Q. (2018). A flyweight and superelastic graphene aerogel as a high-capacity adsorbent and highly sensitive pressure sensor. J. Mater. Chem. A.

[3] Huangfu Y.M., Ruan K.P., Qiu H., Lu Y.J., Liang C.B., Kong J., Gu J.W. (2019). Fabrication and investigation on the pani/mwcnt/thermally annealed graphene aerogel/epoxy electromagnetic interference shielding nanocomposites. Compos. Part. A-Appl. Sci. Manuf..

[4] He C.L., Huang J.Y., Li S.H., Meng K., Zhang L.Y., Chen Z., Lai Y.K. (2018). Mechanically resistant and sustainable cellulose-based composite aerogels with excellent flame retardant, sound-absorption, and superantiwetting ability for advanced engineering materials. ACS Sustain. Chem. Eng..

[5] Chen Y., Zhou L., Chen L., Duan G., Mei C., Huang C., Han J., Jiang S. (2019). Anisotropic nanocellulose aerogels with ordered structures fabricated by directional freeze-drying for fast liquid transport. Cellulose.

[6] Ding Q., Xu X., Yue Y., Mei C., Huang C., Jiang S., Wu Q., Han J. (2018). Nanocellulose-mediated electroconductive self-healing hydrogels with high strength, plasticity, viscoelasticity, stretchability, and biocompatibility toward multifunctional applications. ACS Appl. Mater. Interfaces.

[7] Zhao S.Y., Malfait W.J., Demilecamps A., Zhang Y.C., Brunner S., Huber L., Tingaut P., Rigacci A., Budtova T., Koebel M.M. (2015). Strong, thermally superinsulating biopolymer-silica aerogel hybrids by cogelation of silicic acid with pectin. Angew. Chem. Int. Edit..

[8] Jiang S., Agarwal S., Greiner A. (2017). Low-density open cellular sponges as functional materials. Angew. Chem. Int. Edit..

[9] Hu T., Li L., Zhang J. (2018). Green synthesis of ant nest-inspired superelastic silicone aerogels. ACS Sustain. Chem. Eng..

[10] Prakash S., Brinker C., Hurd A., Rao S. (1995). Silica aerogel films prepared at ambient pressure by using surface derivatization to induce reversible drying shrinkage. Nature.

[11] Gao Y., Zhao S., Zhang G., Deng L., Li J., Sun R., Li y., Wong C.-P. (2015). In situ assembly of dispersed ag nanoparticles on hierarchically porous organosilica microspheres for controllable reduction of 4-nitrophenol. J. Mater. Sci..

[12] Fotoohi B., Kazemzad M., Mercier L. (2018). Additive-free synthesis of robust monolithic mesoporous silica support used in catalysis. Ceram. Int..

[13] Von der Lehr M., Seidler C.F., Taffa D.H., Wark M., Smarsly B.M., Marschall R. (2016). Proton conduction in sulfonated organic-inorganic hybrid monoliths with hierarchical pore structure. ACS Appl. Mater. Interfaces.

[14] Yang Y., Zhang W., Zhang Y., Zheng A., Sun H., Li X., Liu S., Zhang P., Zhang X. (2015). A single au nanoparticle anchored inside the porous shell of periodic mesoporous organosilica hollow spheres. Nano. Res..

[15] Jana A., Mondal J., Borah P., Mondal S., Bhaumik A., Zhao Y. (2015). Ruthenium bipyridyl tethered porous organosilica: A versatile, durable and reusable heterogeneous photocatalyst. Chem. Commun..

[16] Duan G., Bagheri A.R., Jiang S., Golenser J., Agarwal S., Greiner A. (2017). Exploration of macroporous polymeric sponges as drug carriers. Biomacromolecules.

[17] Li L., Hu T., Yang Y., Zhang J. (2019). Strong, compressible, bendable and stretchable silicone sponges by solvent-controlled hydrolysis and polycondensation of silanes. J. Colloid Interface Sci..

[18] Zhang H., Lu J., Peng J., Du G., Peng H., Fang Y. (2018). One-Step preparation of emulsion-templated amino-functionalized porous organosilica monoliths for highly efficient cr(vi) removal. Colloid Surf. A-Physicochem. Eng. Asp..

[19] Velikova N., Vueva Y., Ivanova Y., Salvado I., Fernandes M., Vassileva P., Georgieva R., Detcheva A. (2013). Synthesis and characterization of sol-gel mesoporous organosilicas functionalized with amine groups. J. Non-Cryst. Solids.

[20] Wu C., Liang Y., Yang K., Min Y., Liang Z., Zhang L., Zhang Y., Wu C., Min Y. (2016). Clickable periodic mesoporous organosilica monolith for highly efficient capillary chromatographic separation. Anal. Chem..

[21] Gong G., Nagasawa H., Kanezashi M., Tsuru T. (2018). Facile and scalable flow-induced deposition of organosilica on porous polymer supports for reverse osmosis desalination. ACS Appl. Mater. Interfaces.

[22] Sankaraiah S., Lee J.M., Kim J.H., Choi S.W. (2008). Preparation and characterization of surface-functionalized polysilsesquioxane hard spheres in aqueous medium. Macromolecules.

[23] Kim Y.B., Kim Y.-A., Yoon K.-S. (2006). Preparation of functionalized polysilsesquioxane and polysilsesquioxane-metal nanoparticle composite spheres. Macromol. Rapid Commun..

[24] Dong F., Ha C.S. (2012). Multifunctional materials based on polysilsesquioxanes. Macromol. Res..

[25] Dong F., Guo W., Park S.-K., Ha C.-S. (2012). Controlled synthesis of novel cyanopropyl polysilsesquioxane hollow spheres loaded with highly dispersed au nanoparticles for catalytic applications. Chem. Commun..

[26] Lu L.Y., Li J., Li H.W., Gao C.T., Xie H.B., Xiong Y.Z., Luo Z., Sun Q., Dong F.P. (2019). Controllable synthesis of hierarchical polysilsesquioxane surfaces: From spheres-on-sphere to bowls-on-sphere structure. Appl. Surf. Sci..

[27] Li H., Lu L., Xiong Y., Dong F. (2019). Uniform and reactive hydrogen polysilsesquioxane hollow spheres immobilized with silver nanoparticles for catalytic reduction of methylene blue. Appl. Surf. Sci..

[28] Hayase G., Kanamori K., Fukuchi M., Kaji H., Nakanishi K. (2013). Facile synthesis of marshmallow-like macroporous gels usable under harsh conditions for the separation of oil and water. Angew. Chem. Int. Edit..

[29] Li J., Dong F., Lu L., Li H., Xiong Y., Ha C.-S. (2019). Raspberry-Like polysilsesquioxane particles with hollow-spheres-on-sphere structure: Rational design, controllable synthesis, and catalytic application. Polymers.

[30] Kim Y.H., Choi G.M., Bae J.G., Kim Y.H., Bae B.S. (2018). High-performance and simply-synthesized ladder-like structured methacrylate siloxane hybrid material for flexible hard coating. Polymers.

[31] Zhang L., Jiang F., Chen G.X., Li Q.F. (2009). Synthesis and characterization of mercaptopropyl polyhedral oligomeric silsesquioxane (poss). J. B. Univ. Chem. Technol (Nat. Sci. Ed.).

[32] Lis M.J., Caruzi B.B., Gil G.A., Samulewski R.B., Bail A., Pereira Scacchetti F.A., Moises M.P., Bezerra F.M. (2019). In-situ direct synthesis of hkust-1 in wool fabric for the improvement of antibacterial properties. Polymers.

[33] Bel-Hassen R., Boufi S., Salon M.C.B., Abdelmouleh M., Belgacem M.N. (2008). Adsorption of silane onto cellulose fibers. Ii. The effect of ph on silane hydrolysis, condensation, and adsorption behavior. J. Appl. Polym. Sci..

[34] Guo W., Wang J., Lee S.J., Dong F., Park S.S., Ha C.S. (2010). A general ph-responsive supramolecular nanovalve based on mesoporous organosilica hollow nanospheres. Chem. Eur. J..

[35] Ozawa N., Hayashi K., Yamaura S.-I., Zhang W., Sakamoto W., Yogo T. (2016). Synthesis of inorganic-organic hybrid membranes consisting of triazole linkages formed by the azide-alkyne click reaction. J. Membr. Sci..

[36] Tanaka T., Kanezashi M., Nagasawa H., Tsuru T. (2019). Effects of calcination condition on the network structure of triethoxysilane (tries) and how Si-H groups influence hydrophobicity under hydrothermal conditions. Ind. Eng. Chem. Res..

[37] Martinez E., Plans J., Ynduráin F. (1987). Atomic oxygen in silicon: The formation of the Si-O-Si bond. Phys. Rev. B J..

[38] Wang S.N., Zhang M.C., Zhang W.Q. (2011). Yolk-Shell catalyst of single au nanoparticle encapsulated within hollow mesoporous silica microspheres. ACS Catal..

[39] Peng Y., Leng W., Dong B., Ge R., Duan H., Gao Y.A. (2015). Bottom-up preparation of gold nanoparticle-mesoporous silica composite nanotubes as a catalyst for the reduction of 4-nitrophenol. Chin. J. Catal..

[40] Dong Z., Yu G., Le X. (2015). Gold nanoparticle modified magnetic fibrous silica microspheres as a highly efficient and recyclable catalyst for the reduction of 4-nitrophenol. New J. Chem..

